# Recombinant porcine epidermal growth factor-secreting *Lactococcus lactis* promotes the growth performance of early-weaned piglets

**DOI:** 10.1186/s12917-014-0171-1

**Published:** 2014-08-21

**Authors:** Dingyue Wang, Shengyu Xu, Yan Lin, Zhengfeng Fang, Lianqiang Che, Bai Xue, De Wu

**Affiliations:** 1 Institute of Animal Nutrition, Key Laboratory for Animal Disease Resistance Nutrition of Sichuan Province, Sichuan Agricultural University, Ya’an 625014Sichuan, China

**Keywords:** Porcine epidermal growth factor, Lactococcus lactis, Early-weaned piglets, Intestinal development, Production performance

## Abstract

**Background:**

Epidermal growth factor (EGF) is an important growth factor in regulation of cell proliferation, differentiation, survival and apoptosis. Studies showed that food-grade *Lactococcus lactis* (*L. lactis*) and NICE expression system have superior performance in exogenous protein expression. This study aimed to construct and express porcine EGF (pEGF), and use *L. lactis* as vehicle for producing and delivering pEGF. Furthermore, investigating biological activity of pEGF and exploring applications feasibility of combination effects of *L. lactis* and pEGF on early weaned piglets’ production.

**Results:**

A recombinant *Lactococcus lactis* which produced and secreted pEGF at 1000 ng/ml in culture supernatant was generated. Secreted pEGF was a fully biologically active protein, as demonstrated by its capacity to stimulate L929 mouse fibroblast cell line proliferation *in vitro*. For *in vivo* study, forty piglets were randomly allocated to control, antibiotic control, empty vector-expressing *L. lactis* (LL-EV) and pEGF-secreting *L. lactis* (LL-pEGF). After 14 d of rearing, final body weight and average daily gain in LL-pEGF were greater (*P* < 0.05, 8.95 vs. 8.37 kg, 206.1 vs. 157.7 g/day, respectively) than those in control, but no significant differences between LL-pEGF, LL-EV and antibiotic control. Overall period average daily feed intake was higher in LL-pEGF, LL-EV and antibiotic control than in control (*P* < 0.05, 252.9, 255.6, 250.0, 207.3 g/day, respectively). No significant difference was observed on ADFI/ADG. LL-pEGF increased villous height in the duodenum, jejunum and ileum than in control and LL-EV (*P* < 0.05). Sucrase in the 3 intestinal segments, aminopeptidase A in the duodenum and Jejunum, aminopeptidase N and dipeptidase IV in the duodenum in LL-pEGF were higher than those in control (*P* < 0.05). Furthermore, *Escherichia coli* and *Enterococcus* counts decreased in the ileum and *Lactobacillus* increased in the ileum and cecum digesta in LL-pEGF compare with the control (*P* < 0.05). *Lactobacillus* increased in the cecum in LL-EV compared with control and antibiotic control (*P* < 0.05).

**Conclusion:**

We have generated a recombinant *Lactococcus lactis* which produced and secreted fully biologically active porcine EGF. Oral administration of pEGF-secreting *L. lactis* had beneficial effects on intestinal health and performance of early-weaned piglets.

## Background

Free-ranging domestic piglets start to eat solid food from the age of about 3 weeks, and gradually eat increasing amounts of solid food before weaning at between 13 weeks and 17 weeks when the piglet has free access to its mother, closely resembling that of wild boars and feral pigs [1]-[4]. The weaning time is reported to be earlier in a loose-housing system of about 11 weeks to 12 weeks [5]. Based on information from several experiments and surveys, it has been concluded that early weaning at 3-4 weeks can be an effective management strategy to improve wean-to-finish growth performance and provide a higher number of piglets per sow per year in commercial multi-site production systems which would well control the disease transfer from the sow to the piglet [6]. When weaning at this time, a piglet’s digestive apparatus is undeveloped and an abrupt transition from maternal milk to solid feed entails several problems. This leads to damaged mucosal integrity and reduced enzyme activities, and quite an amount of undigested nutrients are available for enteric pathogenic bacteria, which changes the intestinal microbiota and increases the rate of diarrhea. Although the application of antibiotics alleviated this problem to some extent, antibiotic use induced antibiotic-resistant bacteria generation, resulting in restriction of the use of antibiotics in present animal production [7]-[9]. How to help piglets overcoming post-weaning stress without any side effects are attracting people's attention at the present stage.

Stimulatory effects of colostrums and milk on growth and development of the intestine have long been recognized, and the putative role of milkborne growth factors in mediating this development has been the subject of considerable investigation [10],[11]. Epidermal growth factor (EGF), a small mitogenic polypeptide comprising 53 amino acid residues is typically present at about 1500 ng/ml and 124 ng/ml in porcine colostrum and mature milk, respectively [12]. EGF is synthesized in the salivary glands and kidneys and, to a lesser extent, in the lactating mammary glands, small intestine, liver and pancreas [13]-[15]. EGF has high stability in the gastrointestinal tract of human, mouse, rat and pig, especially in the gastric lumen and the proximal and mid-intestine lumen, with a higher degradation in the distal intestine lumen [16]-[20]. EGF signals via binding to the EGF receptor (EGFR, also known as ErbB1), a transmembrane receptor tyrosine kinase of the ErbB family [21],[22]. Binding of EGF receptor to EGF leads to autophosphorylation of receptor tyrosine kinase and subsequent activation of signal transduction pathways that are involved in regulating cellular survival, growth, proliferation and differentiation [23],[24]. The effects of EGF in stimulating growth of the gastrointestinal tract and inducing small intestinal enzymes have been reported [12],[25]-[27]. EGF was also found to reduce the colonization of the intestinal epithelium by enteropathogens [28].

*Lactococcus lactis* is a food-grade, Gram-positive lactic acid bacterium that, in addition to its traditional use in food fermentations, is increasingly used in modern biotechnological applications. *L. lactis* is a novel cell factory for the production of proteins and bioactive compounds. Using pAMJ399 as an expression vector, which contains a pH-regulated promoter, P170, and an erythromycin-resistance gene for selection, Cheung et al. (2009) found that the transgenic *L. lactis* produced about 0.5 ug EGF per milliliter culture supernatant [29]. However, transcription from P170 was induced between pH 6.5 and pH 6.0, only when the culture entered stationary phase [30]. The protein production ability of *L. lactis* was facilitated by the development of an efficient expression system, such as the Nisin-controlled expression (NICE) system. The NICE system consists of a bacterial host with *nisRK* regulatory genes integrated into the chromosome and an expression vector carrying the gene of interest under the control of a *nisA* promoter [31]. Using this system, expression can be efficiently controlled through the addition of nisin [31]. The *ups45* gene encodes the major extracellular protein Usp45 from *L. lactis* MG1363. Chimeras of the 27-amino-acid Usp45 signal peptide (SP_*usp45*_), fused to a number of mature proteins and peptides, have been used to drive secretion by *L. lactis*, which is metabolically active in all compartments of the intestinal tract. Kuipers et al. (1995) found that lactococcal nisin biosynthesis was autoregulated in *L. lactis*[32]*.* Further study established that exogenous proteins production continued in *L. lactis* 10 h after a 1-h nisin-pulse induction [33]. Therefore, NICE system is an ideal expression system to secrete foreign proteins. The first objective of the present study was to test the hypothesis that porcine EGF can be produced and efficiently secreted in the NICE system by *L. lactis* in a soluble and biologically active form. A second objective was to further determine the effects of oral administration pEGF**-**secreting *L. lactis* whole-cell suspension on intestinal development, digestive enzyme activities, intestinal microbiota and the performance of segregated early weaned pigs, to explore the application feasibility of the effects of microflora and specificity functional protein in the actual production.

## Methods

### Bacterial strains, plasmids and growth media

The expression strain *L. lactis* NZ9000 (NIZO food research B.V., Netherlands) was propagated in M17 (Difco, NJ, USA) containing 0.5% (wt/vol) glucose at 30°C without shaking. *Escherichia coli* (*E. coli*) DH5_α_ strains were grown in Luria-Bertani medium at 37°C under aeration. The expression vector pNZ8148 (NIZO food research B.V., Netherlands) used in *L. lactis* NZ9000 has a chloromycetin-resistance gene for selection. Antibiotics were used when appropriate at the following concentrations: 5 μg/ml chloramphenicol and 50 μg/ml ampicillin.

### Construction of recombinant L. lactis NZ9000/pNZ8148-SPUsp45-MPpEGF

**Figure 1:**
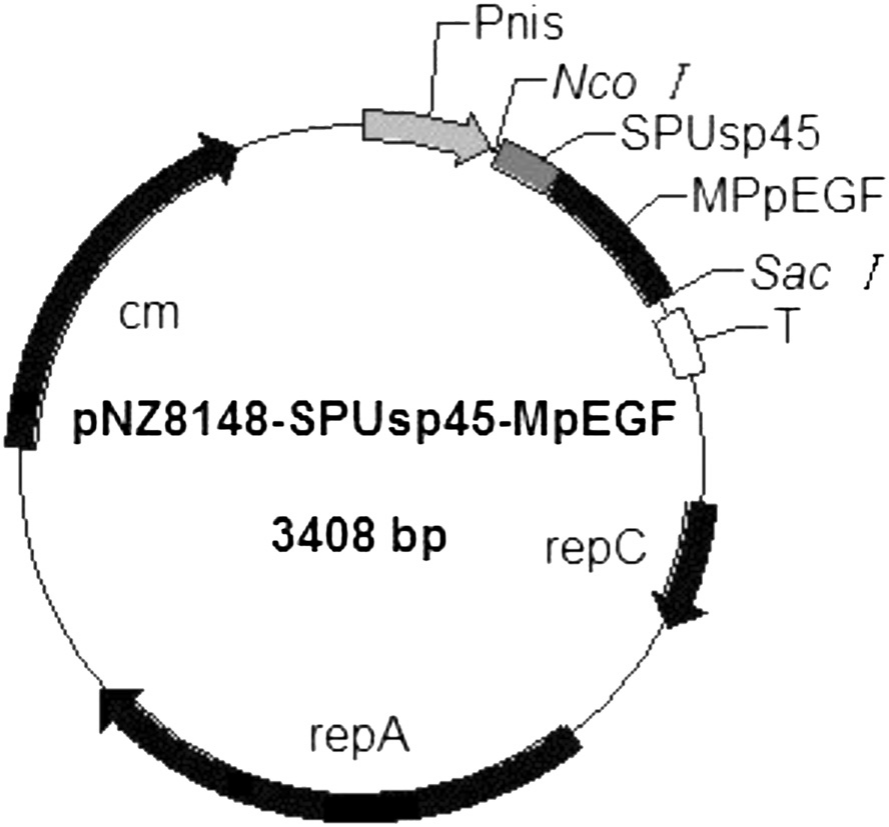
**Schematic representation of pNZ8148-SPUsp45-MpEGF vector expression of MpEGF in*L. lactis*.** A 159-bp DNA fragment encoding mature porcine EGF (MpEGF) was fused in frame with an 81-bp DNA fragment containing the Usp45 signal peptide, derived from the predominant *L. lactis*-secreted protein. In this plasmid, pEGF expression is controlled by the nisin-inducible promoter (P_*nisA*_). The pNZ8148-SPUsp45-MpEGF carries the chloramphenicol resistance marker (cm).

Total RNA was isolated form renal tissue of newborn Meishan piglets using the Trizol (Invitrogen, Carlsbad, CA, USA) method according to the manufacturer’s instructions. Reverse transcription and polymerase chain reactions (PCR) were carried out to amplify the mature pEGF (MPpEGF) sequence (GenBank: NM_214020.1, 2908..3066), using forward primers F1, F2, F3 and reverse primer R. The sequence of the primers were (Bold letters indicate the addition of restriction enzyme sites of *Nco*I and *Sac*I): F1, 5′-GCT GCA GCC CCG TTG TCA GGT GTT TAC GCT AAT AGT TAC TCT GAA TGC C-3′; F2, 5′-CA GCT ATT TTA ATG TCT ACA GTG ATA CTT TCT GCT GCA GCC CCG TTG TCA G-3′; F3, 5′-CAT G**CC ATG G**CT AAA AAA AAG ATT ATC TCA GCT ATT TTA ATG TCT AC-3′ and R: 5′-C **GAG CTC** TTA GCG CAG CTC CCA CCA TTT CAA G-3′. The signal sequence of the major secreted protein Usp45 of *L. lactis subsp. cremoris* MG1363 was contained in the forward primers F1, F2, and F3 with a whole length of the 81 base pairs (GenBank EU382094.1). A unique high fidelity DNA polymerase PrimeSTARTM HS DNA Polymerase (TaKaRa, Osaka, Japan) was used in PCR. The PCR conditions were [98°C (10 sec), 55°C (5 sec), 72°C (1 min)] for 30 cycles. The primers amplified a 259-bp fragment contained 81-bp of SPUsp45 and 159-bp of MPpEGF. TaKaRa LA Taq (Takara, Osaka, Japan) was finally used to amplify the 259-bp DNA fragment with a 3′-A for TA cloning with forward primer F3 and reverse primer R. The 259-bp fusion fragment was verified by 1.2% agarose gel electrophoresis and gel-purified by using an E.Z.N.A.™ Gel Extraction Kit and ligated into pMD19-T Simple Vector to generate pMD19-SPUsp45-MPpEGF. After transforming into *E. coli* DH5_α_ competent cells, positive clones were identified by sequencing, and then pMD19-SPUsp45-MPpEGF was digested with *Nco*I and *Sac*I to release the SPUsp45-MPpEGF insert. The purified insert was subsequently cloned into the corresponding site of expression vector pNZ8148 that was linearized with *Nco*I and *Sac*I. The resultant construct, known as pNZ8148-SPUsp45-MPpEGF, had a sequence of SPUsp45-MPpEGF placed behind the inducible promoter P_nisA_ on the plasmid (Figure 1). The expression construct was then transformed into *E. coli* MC1061; DNA sequencing confirmed the identity of the plasmid. Finally, the plasmid was transformed into *L. lactis* NZ9000 competent cell by electroporation. Recombinant *L. lactis* NZ9000/pNZ8148-SPUsp45-MPpEGF was amplified with primers F0 (F0: 5′-ATAACGCGAGCATAATAAAC-3′) and R and sequenced to select positive recombinant strains. The empty vector pNZ8148 was also transformed into *L. lactis* NZ9000 and was designated as a control (LL-EV).

### Nisin induction

According to the growth and induction condition for *L. lactis* optimized by NIZO, nisin induction of the recombinant *L. lactis* strain was performed as follows. From a culture grown overnight, a 4% (vol/vol) inoculum was transferred to fresh medium and bacteria were grown at 30°C until the optical density at 600 nm was 0.4. Nisin A (Sigma, St Louis, MO, USA) was added at a final concentration of 1 ng/ml, and *L. lactis* cells were propagated for 4 h to 24 h before harvesting.

### Western blot analysis

Western blotting was performed as described [34]. Proteins in cell lysates and supernatant fluids were separated on 15% sodium dodecyl sulfate polyacrylamide gels and then transferred to polyvinylidene fluoride (PVDF; Millipore, Billerica, MA, USA) membranes. The blots were blocked in skim milk-phosphate-buffered saline (PBS) and then incubated with primary anti-EGF (1:500 dilution; Cell Sciences, Canton, MA), followed by incubation with a secondary antibody (anti-rabbit IgG horseradish peroxidase-linked anti body, 1:2000; Cell Signaling Technology, MA, USA). Proteins were detected by the enhanced chemiluminescence (ECL) Advance Western Blotting Detection Kit (Amersham, Piscataway, NJ, USA). Densitometric analyses of band intensities were performed using GeneTools (Syngene, Frederick, MD, USA). Each experiment was repeated at least three times.

### In vitro cell proliferation assay

A L929 mouse fibroblast cell line (ATCC Cat. No. CCL-1) was used in an *in vitro* cell proliferation assay. Cells were seeded in a 6-well culture plate at an initial density of 0.16 × 10^5^ cells/well with 2.5 ml Dulbecco′s Modified Eagle′s Medium (DMEM) containing 100 IU/ml penicillin sodium, 100 μg /ml streptomycin sulfate and 10% fetal bovine serum (FBS). Cells were incubated at 37.0°C in 5% carbon dioxide until 60% confluent. Culture medium was removed and cells were washed 3 times with D-Hanks solution. Subsequently, the cells were incubated in DMEM for 24 h to achieve serum deprivation. Then cells were washed twice with D-Hanks and fresh DMEN medium without FBS and were used for further culture. Cells were treated immediately with the following groups: recombinant human EGF (rhEGF; Cell science; 20 ng/ml), supernatant fluid collected from 24 h nisin A induced *L. lactis* NZ9000/pNZ8148–SPUsp45-MPpEGF (LL-pEGF; 10 or 20 μl), and supernatant fluid collected from *L. lactis* NZ9000/pNZ8148 cultured at the same time as the control (LL-EV; 20 μl). All the supernatants from the cultures were filtered by using 0.22-μm sterile syringe filters before being added into the cell culture medium. After 24 h treatment, cells were trypsinized and quantified using a hemocytometer. Cell counting was done in a blinded-manner by a 3rd party that was not aware of the experimental treatment.

### Piglets and diet treatment

Forty-five crossbred piglets (Duroc × Landrace × Yorkshire) weaned at 21 days were obtained from New Hope Group (Chengdu, SC). After two days of transition feeding, 40 piglets were selected and assigned to 1 of the 4 treatment groups based on sex and body weight: control (M17 broth), antibiotic control (kitasamycin, 25 mg/kg diet), LL-EV (empty vector control, no pEGF was detected) and LL-pEGF (1.8 ug/ml pEGF, the concentration calculated based on the supernatant fluids and the *L. lactis* cell lysates), and each treatment had 10 replicates (5 males and 5 females). All the piglets were fed separately in a metabolic cage with a nipple waterer and a stainless steel feeder for a 14 d experimental period. The piglets were provided *ad libitum* access to water and diet. The basal diet represented a typical commercial corn-soybean meal that met the nutrient requirements suggested by the National Research Council (1998). The diets (Table 1) did not contain any feed antibiotics except for the diet used for piglets in antibiotic control group. Piglets were reared in an environmentally regulated nursery house in Sichuan Agricultural University. Food intake of each piglet was recorded every day and bodyweight was recorded every 7 d. The study was conducted according to the guidelines from the Chinese Council of Animal Care and was approved by the Sichuan Agricultural University Animal Care Committee and all *in vivo* experiments in accordance with the ARRIVE guidelines.

**Table 1:** **Diet composition and nutrient levels (90**% **dry matter basis, %)**

**Item**	
*Ingredients (g/kg)*	
Corn	220.00
Extruded corn	260.00
Dehulled soybean meal	135.00
Full fat extruded soybean	80.00
Fish meal	40.00
Soybean protein concentrate	50.00
Whey	150.00
Sucrose	20.00
Glucose	15.00
Calcium Carbonate	8.00
Calcium hydrophosphate	6.80
L-Lysine	3.20
DL-Methionine	2.00
L-Tryptophan	0.50
L-Threonine	1.50
Vitamin premix^a^	0.40
Trace mineral premix^b^	3.60
Choline chloride	1.00
Salt	3.00
Total	1000.00
*Calculated nutritive composition*	
Digestible energy (KJ/kg)	14460
Crude protein (%)	20.27
Crude fat (%)	3.70
Lysine (%)	1.33
Met + Cys (%)	0.75
Calcium (g/kg)	8.40
Phosphorous (g/kg)	6.30

^a^Supplied per kilogram diet: Vitamin A, 2, 800 IU; Vitamin D_3_ 320 IU; Vitamin E, 20 IU; Vitamin K_3_, 1 mg; Vitamin B_1_, 1.5 mg; Vitamin B_2_, 5 mg; Vitamin B_6_, 2 mg; Vitamin B_12_, 0.03 mg; Nicotonic, 20 mg; Pantothenic, 15 mg; Folic acid, 0.5 mg; Biotin, 0.1 mg.

^b^Supplied per kilogram diet: Cu 6 mg, Fe 100 mg, Mn 4 mg, Zn 100 mg, I 0.14 mg, Se 0.3 mg, Cr 0.2 mg.

LL-pEGF was induced for 24 h as described above, and at the same time LL-EV was harvested and used for the treatments of LL-pEGF and LL-EV. Bacterial cultures were delivered to piglets as a whole-cell suspension. All the piglets were orally fed 50-ml doses, via intragastric gavage, twice daily through a human use stomach tube at 08:00 and 15:00. The piglets in the treatment of control were orally administrated 50-ml sterile M17 broth, and the piglets in the antibiotic control treatment were fed with 50-ml physiological saline in the same manner to create the same stress produced by gavage with other treatments.

### Intestinal sample collection

At the end of the experiment, randomly selected six piglets (3 males and 3 females) from each treatment were anaesthetized with an intravenous injection of sodium pentobarbital [25 mg/kg body weight (BW)] and bled by exsanguinations. The piglet abdomen was opened immediately from the sternum to the pubis; the entire intestine was rapidly removed. A 15 cm section of distal duodenum, mid-jejunum and mid-ileum were ligated and removed to avoid chyme admixture in different intestinal sections. The small intestine was placed on a chilled stainless steel tray. A small incision was opened in the distal end of each segment, and the chyme was collected in a 5 ml tube and stored at 4°C. Two segments of 2 cm and 10 cm were cut at the distal duodenum, mid-jejunum and mid-ileum. The 2 cm intestinal segments were flushed gently with ice-cold PBS (pH 7.4) and then placed in 10% fresh, chilled formalin solution for histological measurement. Intestinal segments (10 cm in length) were opened longitudinally and the contents were flushed with ice-cold PBS. Mucosa was collected with scraping on a sterile glass microscope slide, which was placed on an ice-cold tray, rapidly frozen in liquid nitrogen and stored at -80°C until analysis. Intestinal sections used for histological measurement or mucosa collection were sampled at the same location of each piglet. All samples were collected within 20 min after being killed.

### Intestinal morphology

Formalin-fixed duodenum, jejunum and ileum samples were embedded in paraffin. Cross sections of the segments were cut with a microtome to be approximately 5-μm thick and were stained with hematoxylin and eosin. At least 4 sections of each of the small intestinal segments were checked, and more than 6 complete villous-crypt structures were examined under a microscope in each section. The villus height and the associated crypt depth were measured using a microscope with a Leica Q500MC computer-assisted morphometric system. Villus height was defined as the distance from the villus tip to the crypt mouth, and crypt depth from the crypt mouth to the base.

### Brush border enzyme activity

The enzymes studied were lactase (EC 3.2.1.23), sucrase (EC 3.2.1.48), maltase (EC 3.2.1.20), aminopeptidase A (EC 3.4.11.1), aminopeptidase N (EC 3.4.11.2) and dipeptidyl peptidase IV (EC 3.4.14.5). All assays were carried out on homogenates of mucosal tissue obtained by thawing approximately 1 g of tissue and homogenizing it in 9 ml PBS (pH = 7.2) using an ultrasonic homogenizer. The sample was stored at -80°C before being assayed. Lactase, sucrase and maltase activities were assayed as described by Petersen et al. [35]. The protein content in mucosal homogenates was measured according to Lowry et al. [36], using bovine serum albumin (A 7638, Sigma Chemical, St. Louis) as the standard.

A porcine aminopeptidase A (APA) ELISA Kit (R&D Systems Inc., MN, USA), porcine aminopeptidase N (APN) ELISA Kit (R&D Systems Inc., MN, USA) and porcine dipeptidase IV (DPP-IV) ELISA Kit (R&D Systems Inc., MN, USA) were used for quantitative detection of APA, APN and DPP-IV. Enzymes were first combined to the solid-phase antibody, and then the combined antibody, which was labeled with horseradish peroxidase (HRP) to form an antibody-antigen-enzyme-antibody complex. 3,3′,5,5′-Tetramethylbenzidine (TMB), was used as substrate to be enzyme-catalyzed by HRP. The reaction was terminated by addition of a sulphuric acid solution and measured spectrophotometrically at a wavelength of 450 nm. The enzyme activities were expressed as units per milligram or nanogram per gram of mucosa.

### Intestinal microbiota assay

The microbiological assay of ileum and cecum digesta was carried out according to the procedure of Torrallardona et al. [37]. The digesta collected from the ileum and cecum was homogenized in a sterile salt solution (NaCl 0.85%). Bacterial quantifications for homogenized liquid were made using serial 10 fold dilutions in the sterile salt solution (NaCl 0.85%), followed by plating on an agar medium. The samples of ileal and cecal digesta were cultured in different media for the determination of *E. coli* (MacConkey agar; Merck, Darmstadt, Germany), *Enterococcus* (EC agar; Merck, Darmstadt, Germany), *Bifidobacterium* (BL agar; Difco, NJ, USA) and *Lactobacillus* (MRS agar; Oxoid, Hampshire, UK). The concentration of bacteria in the original sample was calculated as the mean of three dilutions for each sample. The results are expressed as the mean of log_10_ colony forming units (cfu)/g digesta from the 6 piglets with the standard deviations.

### Statistical analyses

**Figure 2:**
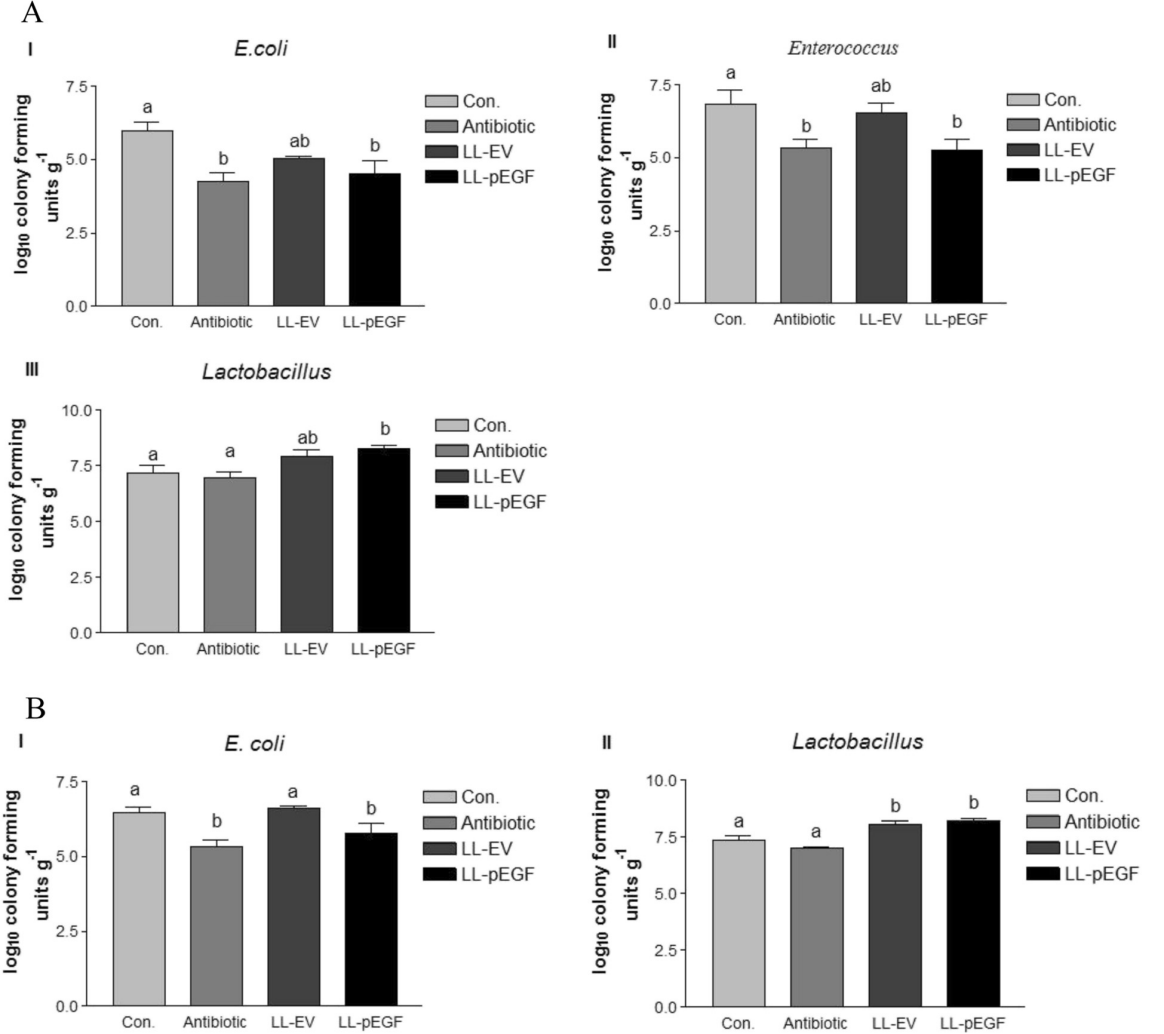
**Effects of different treatments on the ileum microbiota (A) and the cecum microbiota (B) of early-weaned piglets. A**(I), *E. coli* quantification in the ileum; **A**(II), *Enterococcus* quantification in the ileum; **A**(III), *Lactobacillus* quantification in the ileum. **B**(I), *E. coli* quantification in the cecum; **B**(II), *Lactobacillus* quantification in the cecum. Results are expressed as the mean of log_10_ colony forming units (cfu)/g digesta with standard deviations. Different lowercase letters denote statistical differences between groups (*P* < 0.05). Con., control; Antibotic, antibotic control; LL-EV, empty vector-expressing *Lactococcus lactis*; LL-pEGF, pEGF-secreting *Lactococcus lactis*.

Western blot and *in vitro* cell proliferation assays were repeated at least three times, with data representing the mean values with standard deviations of all repeats within an individual experiment. For the animal trial, the experimental model used was a completely randomised design with four treatments and the individual piglets were the experimental units, with data representing the mean values with their standard deviations of all experimental units. Data were analyzed by one-way analysis of variance (ANOVA) followed by either the Bonferroni test for selected pair comparisons [Table 2, final weight, average daily gain (ADG) of second week, average daily feed intake (ADFI) of first week and overall period; Figure 2A (II), the *Enterococcus* quantification; Figure 2B [I], the *E. coli* quantification], or the Tukey test for multiple comparisons (all other tables and figures) for ANOVA to determine statistical differences between groups using GraphPad Prism analysis software. Results were considered significant at *P* < 0.05.

**Table 2:** Effects of diets supplemented with different additives on growth performance of early-weaned pigs (Mean values and standard deviations)

	**Control**	**Antibiotic control**	**LL-EV**	**LL-pEGF**
Initial weight (kg)	6.16 ± 0.38	6.27 ± 0.13	6.09 ± 0.19	6.06 ± 0.31
Final weight (kg)	8.37 ± 0.43^a^	8.91 ± 0.37^ab^	8.68 ± 0.36^ab^	8.95 ± 0.42^b^
ADG (g/day)				
0-7 d	135.7 ± 21.8^a^	142.9 ± 31.9^ab^	172.5 ± 30.4^ab^	178.6 ± 26.7^b^
8-14 d	179.6 ± 27.4^a^	230.3 ± 42.4^ab^	203.6 ± 33.3^ab^	233.7 ± 44.9^b^
0-14 d	157.7 ± 25.4^a^	188.6 ± 31.1^ab^	184.3 ± 29.5^ab^	206.1 ± 27.1^b^
ADFI (g/day)				
0-7 d	168.1 ± 31.7^a^	191.1 ± 44.3^ab^	215.9 ± 28.4^b^	217.1 ± 40.1^b^
8-14 d	246.6 ± 31.1	300.8 ± 38.0	295.5 ± 52.9	288.6 ± 34.9
0-14 d	207.3 ± 27.0^a^	250.0 ± 37.1^b^	255.6 ± 33.3^b^	252.9 ± 34.2^b^
ADFI/ADG				
0-7 d	1.24 ± 0.12	1.39 ± 0.11	1.31 ± 0.30	1.22 ± 0.15
8-14 d	1.41 ± 0.32	1.31 ± 0.20	1.45 ± 0.04	1.26 ± 0.21
0-14 d	1.34 ± 0.25	1.34 ± 0.14	1.39 ± 0.15	1.23 ± 0.12

N = 10.

LL-EV, empty vector-expressing *Lactococcus lactis* group; LL-pEGF, pEGF-secreting *Lactococcus lactis* group; ADG, Average daily gain; ADFI, Average daily feed intake.

^a,b^Means with different superscripts in the same row indicate significant differences (*P* < 0.05).

## Results

### Characterization of pEGF production by L. lactis

**Figure 3:**
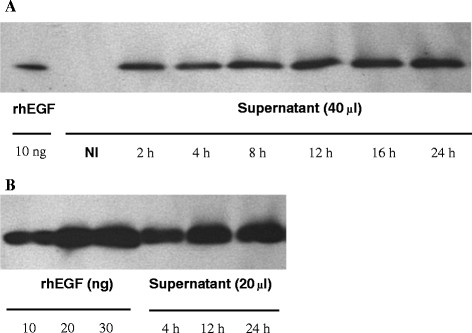
**Quantification of porcine EGF secreted by*L. lactis*. A)** Western blot analysis shows that the 6.0-kD porcine EGF was present in the supernatant of the induced LL-pEGF fermentation cultures, and no secreted pEGF was detected in the supernatant of the non-induced LL-pEGF fermentation cultures (NI). **B)** A representative western blot of supernatants from the induced LL-pEGF. Supernatants of the LL-pEGF induced at different times were used to determine the amounts of pEGF present in the culture supernatant by densitometry from three experiments. The pEGF concentrations in the LL-pEGF culture were about 600 ng/ml, 900 ng/ml and 1000 ng/ml at 4 h, 12 h, and 24 h, respectively. rhEGF, recombinant porcine epidermal growth factor; NI, non-induced LL-pEGF fermentation cultures.

**Figure 4:**
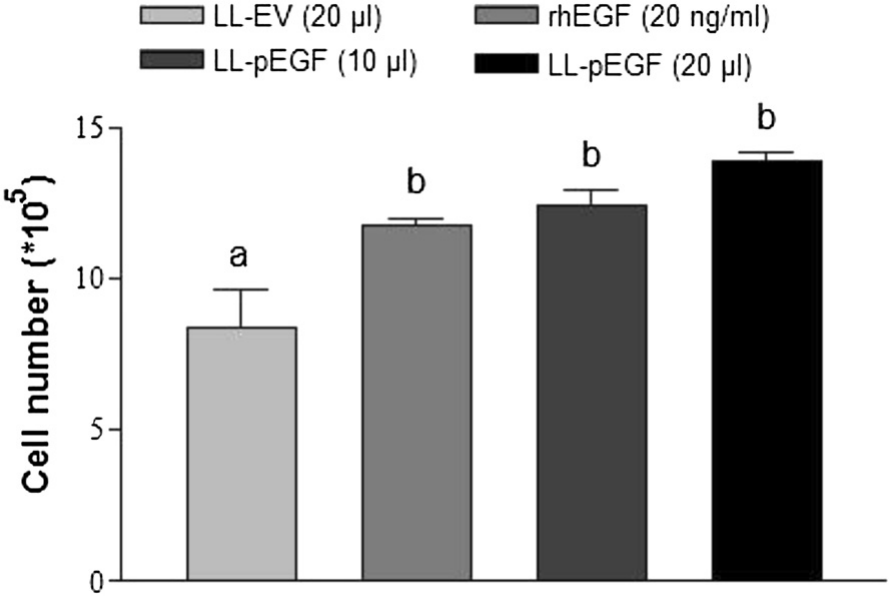
***In vitro*biological activity of porcine EGF produced by*L. lactis*.** Twenty-four hours after serum deprivation, L929 mouse fibroblast cells were cultured for an additional 24 h in 2.0 ml of DMEM containing 20 ng/ml of rhEGF, 20 μl of supernatant from LL-EV, and 10 μl or 20 μl of supernatant from LL-pEGF. Results are expressed as the cell numbers of each treatment and represent the means of three independent experiments performed in triplicate. Different lowercase letters denote statistical differences between groups at *P* < 0.05. rhEGF, recombinant porcine epidermal growth factor; LL-EV, empty vector-expressing *Lactococcus lactis*; LL-pEGF, pEGF-secreting *Lactococcus lactis*.

The secreted porcine EGF bands of 6.0 kDa were detected in nisin-induced cultures of the LL-pEGF strain, and no signal was detected in non-induced LL-pEGF cultures by western blotting (Figure 3A). The concentration of porcine EGF in the recombinant bacterial culture supernatant collected after 24 hours of incubation was 1000 ng/ml based on comparative densitometry analysis of the bands with that of a known amount of rhEGF (Figure 3B). Treatment of fibroblasts with supernatant (10 μl or 20 μl) from the LL-pEGF culture significantly (*P* = 0.003) increased cell numbers during a 24-h culture compared with the control group in which 20 μl of the supernatant from the LL-EV culture was added (Figure 4). Recombinant pEGF secreted by *L. lactis* had the same biological activity as rhEGF for stimulating cell proliferation (Figure 4).

### Growth performance of early-weaned piglets

The effects of dietary treatments on growth performance of early-weaned piglets were illustrated in Table 2. LL-pEGF group had higher final BW compare with the control group (*P* = 0.044). Overall ADG (from 0 to 14 d, *P* = 0.031), ADG of the first week (*P* = 0.019) and second week (*P* = 0.043) in LL-pEGF group were greater than in the control group. No significant differences were observed in final BW and ADG among LL-pEGF, antibiotic control and LL-EV groups. ADFI of first week was higher in LL-pEGF and LL-EV than in the control group (*P* = 0.042). The overall period ADFI were higher in LL-pEGF, LL-EV and antibiotic control than in control group (*P* = 0.036). No differences were observed on feed efficiency (expressed as ADFI/ADG).

### Intestinal development and brush border enzyme activity

As shown in Table 3, the length of villi in the duodenum (*P* = 0.001), jejunum (*P* < 0.001) and ileum (*P* < 0.001) was longer in the LL-pEGF treatment group than in control and LL-EV groups. Antibiotic treatment showed higher villi heights in the ileum than in controls (*P* < 0.001). In terms of crypt depth, there was no significant difference among the treatment groups at the duodenum, jejunum and ileum.

**Table 3:** Effects of diets supplemented with different additives on intestinal development of early-weaned pigs (Mean values and standard deviations)

	**Control**	**Antibiotic control**	**LL-EV**	**LL-pEGF**
Villus height (μm)				
Duodenum	275.59 ± 39.77^a^	299.25 ± 27.03^ab^	292.62 ± 19.30^a^	332.20 ± 40.82^b^
Jejunum	272.34 ± 39.69^a^	312.55 ± 47.44^ab^	282.95 ± 30.49^a^	345.07 ± 29.75^b^
Ileum	216.90 ± 25.54^a^	271.93 ± 39.91^bc^	235.29 ± 30.10^ab^	279.38 ± 41.66^c^
Crypt depth (μm)				
Duodenum	230.71 ± 35.98	203.29 ± 19.75	209.89 ± 24.81	205.78 ± 30.47
Jejunum	202.51 ± 31.19	189.19 ± 22.81	191.98 ± 26.32	181.51 ± 28.46
Ileum	159.64 ± 24.57	143.58 ± 23.97	148.37 ± 28.10	143.70 ± 22.71

N = 6.

LL-EV, empty vector-expressing *Lactococcus lactis* group; LL-pEGF, pEGF-secreting *Lactococcus lactis* group.

^a,b,c^Means with different superscripts in the same row indicate significant differences (*P* < 0.05).

Brush border enzyme activities are shown in Table 4. No difference was observed in brush border lactase activity in the duodenum, jejunum and ileum among the treatments. Duodenum and jejunum sucrase activities in LL-pEGF group were the same as that in antibiotic control and LL-EV groups, but they were greater than that in controls group (*P* < 0.05). Ileum sucrase activity in LL-pEGF group was greater (*P* = 0.018) than that in antibiotic control group, control group and LL-EV group. No differences were observed of maltase activity in the duodenum and the jejunum among treatment groups, but that in the ileum was greater (*P* < 0.05) in LL-pEGF group than in antibiotic control group and LL-EV group. The activity of APA, APN and DPP-IV were increased in the duodenum compared with controls and the antibiotic controls (*P* < 0.05). The activity of APA also was increased in the LL-pEGF treatment group compared with the controls and LL-EV treatment group in the jejunum (*P* < 0.05). There were no significant differences among treatments in the activities of APA in the ileum and APN and DPP-IV in the jejunum and ileum (Table 4).

**Table 4:** Effects of diets supplemented with different additives on brush border enzyme activity of early-weaned piglets (Mean values and standard deviations)

	**Control**	**Antibiotic control**	**LL-EV**	**LL-pEGF**
Lactase (U/mg)				
Duodenum	2.73 ± 0.75	2.07 ± 0.98	1.90 ± 0.29	2.63 ± 0.63
Jejunum	1.32 ± 0.27	1.37 ± 0.48	1.56 ± 0.53	1.24 ± 0.33
Ileum	0.43 ± 0.19	0.45 ± 0.22	0.36 ± 0.09	0.40 ± 0.11
Sucrase (U/mg)				
Duodenum	0.69 ± 0.43^a^	0.99 ± 0.46^ab^	1.92 ± 1.35^ab^	2.84 ± 1.00^b^
Jejunum	3.12 ± 2.28^a^	5.83 ± 2.58^ab^	7.03 ± 0.87^ab^	9.49 ± 2.28^b^
Ileum	8.91 ± 0.42^a^	9.08 ± 1.51^a^	9.13 ± 0.95^a^	11.33 ± 0.93^b^
Maltase (U/mg)				
Duodenum	9.19 ± 1.35	7.17 ± 1.76	10.26 ± 2.45	12.07 ± 3.91
Jejunum	3.78 ± 1.89	4.09 ± 1.61	2.43 ± 0.78	4.74 ± 1.77
Ileum	6.93 ± 1.10^ab^	4.21 ± 1.42^a^	4.77 ± 1.45^a^	10.18 ± 3.30^b^
APA (ng/g)				
Duodenum	23.21 ± 2.16^a^	20.89 ± 2.64^a^	25.96 ± 4.33^ab^	35.19 ± 7.52^b^
Jejunum	23.24 ± 1.30^a^	29.68 ± 1.31^ab^	23.39 ± 5.38^a^	35.20 ± 4.35^b^
Ileum	33.69 ± 7.22	33.74 ± 2.07	32.23 ± 3.82	33.20 ± 5.83
APN (ng/g)				
Duodenum	8.33 ± 1.44^a^	6.96 ± 0.68^a^	9.98 ± 1.27^ab^	12.33 ± 2.70^b^
Jejunum	9.27 ± 1.81	9.61 ± 2.83	10.42 ± 1.46	12.44 ± 2.25
Ileum	11.23 ± 2.53	12.15 ± 1.62	11.61 ± 2.01	14.84 ± 3.68
DPP-IV (ng/g)				
Duodenum	8.37 ± 1.11^a^	6.99 ± 0.87^a^	9.22 ± 1.39^ab^	11.89 ± 2.47^b^
Jejunum	10.03 ± 1.92	9.73 ± 0.86	8.34 ± 2.27	9.15 ± 2.44
Ileum	10.32 ± 1.97	10.65 ± 0.91	10.44 ± 1.28	13.35 ± 1.78

N = 6.

LL-EV, empty vector-expressing *Lactococcus lactis* group; LL-pEGF, pEGF-secreting *Lactococcus lactis* group; APA, aminopeptidase A; APN, aminopeptidase N; DPP-IV, dipeptidase IV.

^a,b^Means with different superscripts in the same row indicate significant differences (*P* < 0.05).

### Intestinal microbiota

The number of *E. coli* in the ileum in LL-pEGF group was similar (*P >* 0.05) to that in antibiotic control group, lower (*P* = 0.01) than that in control group (Figure 2A [I]). LL-pEGF and antibiotic treatments reduced the number of *Enterococcus* (*P* = 0.022, Figure 2A [II]) in the ileum. There was no significant difference observed in *Bifidobacterium* among the treatments (data not shown) in the ileum. Oral administration of *L. lactis-*secreting pEGF resulted in an increase of 1.09 log_10_ cfu/g and 1.31 log_10_ cfu/g in the number of *Lactobacillus* in the ileal digesta compared with controls and antibiotic treatments, respectively (*P* < 0.05, Figure 2A [III]).

For the microbiota in the cecum, compared with the control and LL-VE treatments, antibiotic treatment and LL-pEGF decreased the number of *E. coli* (*P =* 0.004, Figure 2B [I]). No significant difference was observed in *Enterococcus* and *Bifidobacterium* among the treatments (data not shown) in the cecum. Oral administration of *L. lactis-*secreting pEGF increased the amount of *Lactobacillus* in the cecum by 0.82 log_10_ cfu/g and 1.19 log_10_ cfu/g compared with the control and antibiotic treatments, respectively (*P <* 0.001, Figure 2B [II]). LL-VE treatment also increased the amount of *Lactobacillus* in the cecum compared with the control and antibiotic treatments, respectively (*P <* 0.001, Figure 2B [II]).

## Discussion

Early weaning has been used extensively to shorten interval of farrowing thus improve litter size per year and control the transfer of disease from the sow to the piglet, but the weaning stress was consequently intensified in piglets, which induced an intestinal villi height decrease and crypt depth increase, and enhanced the rate of diarrhea [38]-[40]. The problem of piglets overcoming post-weaning stress has plagued people for a long time. The application of antibiotics do played an important role at this problem, but it also has the limitations due to the antibiotics resistance [7],[8]. The current study demonstrated that supplementing diets with pEGF-secreting *L. lactis* (a swine-derived growth factor and the probiotics) facilitates intestinal health in early-weaned piglets, which might be a new way to help the animals overcome the weaning transition.

In this work, we cloned the porcine *egf* gene into a nisin-controlled expression vector, resulting in a vector that allows rapid and efficient secretion of soluble pEGF by *L. lactis*. Western blot analyses showed that the concentrations of pEGF proteins were increased in the supernatant of the induced LL-pEGF fermentation cultures that were collected at different time points. The peak occurred at 24 h after nisin was induced (no significant differences in concentrations of pEGF proteins collected at 36 h and 48 h compared with 24 h, data not shown). The concentration of pEGF from our nisin-controlled expression vector was higher than that from the pAMJ399 expression vector (1000 ng/ml vs. 500 ng/ml) [29]. We demonstrated that our recombinant pEGF is directly secreted in a biologically active form, as shown by its capacity to efficiently stimulate L929 mouse fibroblast cell proliferation. pEGF production in a secreted form allows for direct assessment of its biological activity in small volumes of culture medium, without any purification steps. This procedure was easily adapted for large-scale production of biologically active recombinant pEGF.

The role of EGF in stimulating intestinal epithelium proliferation, differentiation and intestinal maturation has been demonstrated [27],[41],[42]. EGF implements its function through binding to EGFR. Chandra and his colleagues demonstrated that activation of EGFR increases the cell number by promoting cell proliferation and suppressing either serum depletion-induced or TNFα-induced apoptosis [43]. EGFR was found located on the basolateral surfaces in the infant and adult gastrointestinal tract [21],[44]. In weaned pigs, EGFR appeared on both microvillar and basolateral surfaces of enterocytes. In the duodenum, EGFR was evenly distributed in the villi and crypts and present to a lesser extent in the duodenal glands. In the jejunum, most EGFR was seen in the top of the villi. In the ileum, most EGFR was seen to decrease from the villus tip to the crypt [45]-[47]. Furthermore, it has been suggested that there are 10 times more EGF receptors than EGF itself, which suggests that exogenous EGF administration may be taken up and well used by animals [48]. Consistent with previous reports, this study demonstrated that our recombinant pEGF stimulates L929 mouse fibroblast cell proliferation *in vitro* and increases mean villus height *in vivo.*

In the *in vivo* study, LL-pEGF improved final BW and ADG compare with the control group, but its effects were similar with the antibiotic control and LL-EV group. The overall period ADFI were higher in LL-pEGF, LL-EV and antibiotic control groups than in the control group. The higher BW gain in LL-pEGF pigs could be resulting from the adequate intake of nutrients, as indicated by the markedly increased ADFI in LL-pEGF pigs than in control pigs. However, antibiotic and LL-EV supplementation were able to exert a growth rate similar to LL-pEGF when receiving a similar feed intake.

The Oral administration of LL-pEGF to early-weaned piglets enhanced intestinal development by increased villi height in the three intestinal segments compared with control and LL-EV group in this study. This is consistent with previous reports regarding the possible promotion of proliferation of EGF [26],[27],[49]-[51]. The antibiotics were helpful to increasing the villi height compared with the negative control in piglets [52]-[54]. In the present study, villi height of the antibiotic group was increased compared with control and were similar to LL-pEGF in the ileum. The increased villous height implied the increased surface area for nutrient absorption [55], thus may have resulted in higher growth rate in the LL-pEGF treatment than others.

Diets supplemented with the recombinant EGF from *Pichia pastoris* strain elevated jejunal alkaline phosphatase, sucrase, lactase and maltase activities in early-weaned piglets [56]. In newborn and weaned piglets, systemic or oral administration of EGF significantly increased jejunal lactase and sucrase activities [12],[25], suggesting EGF modulates enterocyte differentiation. It also reported that EGF plays an important role in cell differentiation rather than cell proliferation in young animals, and it may be involved in stimulating mucus secretion [46]. Our findings that LL-pEGF increases mean sucrase activities in the 3 intestinal segments compared with control, maltase activities in the ileum compared with antibiotic control and LL-EV group, APA, APN and DPP-IV in the duodenum compared with control and antibiotic control are consistent with these reports. The elevation of intestinal enzyme activity in LL-pEGF should be one reason to result in improvement of growth performance in this study.

In addition, the intestinal microbiotas interact with host intestinal epithelial cells and protect them from pathogens via interference with pathogen adhesion and invasion [57]. They also affect the host through the immune response and the metabolic products of fermentation processes. Thus, the intestinal microbiota is very important for the maintenance and recovery of intestinal health. An impaired balance of the intestinal microbiota could have many adverse effects on the health of the host [58]. *Lactobacillus* has been shown to play a role in the inhibition of pathogens in the intestine [59]. Here, we found that both LL-pEGF and LL-EV treatments increased the amount of *Lactobacillus* which suggests that part of the beneficial effects of LL-pEGF can be attributed to the probiotic properties of *L. lactis*. Although EGF did not affect the proliferation of *E. coli in vitro*, it is reported that orally administered EGF protects the gastrointestinal tract against colonization by enteropathogenic *E. coli* in rabbits [28]. Consistent with previous studies, we found LL-pEGF and antibiotic control treatments decreased the amount of *E. coli* in the ileum and cecum, and *Enterococcus* counts in the ileum. It’s well known that the antibiotics would inhibit harmful bacteria, but also the probiotics, and it’s well demonstrated in the present study that the antibiotic control decreased the amount of *E. coli*, *Enterococcus* and *Lactobacillus* which would not conducive to the balance of intestinal microbiota even affect the growth performance of the pig in the subsequent growth stage*.* However, LL-pEGF ameliorated the intestinal microbial flora by increasing the counts of probiotics *L. lactis* and inhibited potential harmful bacteria *E. coli* and *Enterococcus*, thus promoted intestinal health.

## Conclusions

In summary, we have successfully designed a *L. lactis* strain for highly efficient secretion pEGF, which can be directly recovered from the culture medium. The *in vitro* study showed that the secreted pEGF was a fully biologically active protein. The results obtained from the *in vivo* study indicated that oral administration of recombinant pEGF-secreting *L. lactis* promotes the health of the intestine and growth rate in early-weaned piglets. The potential benefits of pEGF-secreting *L. lactis* in weaning transition stage should make it acceptable as an alternative therapy for farm animals during specific stages of development.

## Abbreviations

EGF: Epidermal growth factor

rpEGF: Recombinant porcine EGF

LL-pEGF: pEGF-secreting *Lactococcus lactis*

LL-EV: Empty vector-expressing *Lactococcus lactis*

EGFR: ErbB1 EGF receptor

rhEGF: recombinant human EGF

APA: Aminopeptidase A

APN: Aminopeptidase N

DPP-IV: Dipeptidase IV

cfu: Colony-forming unit

## Competing interests

The authors declare that they have no competing interests.

## Authors’ contributions

The authors’ contributions were as follows: DYW, SYX and DW designed the experiments; DYW and SYX performed the study and wrote the manuscript; YL, ZFF, LQC and BX assisted in writing the manuscript. All authors read and approved the final manuscript.

## References

[B1] Arey D Brooke P Animal Welfare Aspects of Good Agricultural Practice: Pig Production 2006 Compassion in World Farming Trust, Petersfield, UK

[B2] Jensen P Maternal behaviour and mother-young interactions during lacation in free-ranging domestic pigs Appl Anim Behav Sci 1988 20 297 308 10.1016/0168-1591(88)90054-8

[B3] Jensen P Recén B When to wean — observations from free-ranging domestic pigs Appl Anim Behav Sci 1989 23 49 60 10.1016/0168-1591(89)90006-3

[B4] Jensen P Stangel G Behaviour of piglets during weaning in a seminatural enclosure Appl Anim Behav Sci 1992 33 227 239 10.1016/S0168-1591(05)80010-3

[B5] Bøe K The process of weaning in pigs: when the sow decides Appl Anim Behav Sci 1991 30 47 59 10.1016/0168-1591(91)90084-B

[B6] Main RG Dritz SS Tokach MD Goodband RD Nelssen JL Increasing weaning age improves pig performance in a multisite production system J Anim Sci 2004 82 5 1499 1507 15144093 10.2527/2004.8251499x

[B7] Doyle ME Hartmann FA Lee Wong AC Methicillin-resistant staphylococci: implications for our food supply? Anim Health Res Rev 2012 13 2 157 180 10.1017/S1466252312000187 23253164

[B8] Hiroi M Kawamori F Harada T Sano Y Miwa N Sugiyama K Hara-Kudo Y Masuda T Antibiotic resistance in bacterial pathogens from retail raw meats and food-producing animals in Japan J Food Prot 2012 75 10 1774 1782 10.4315/0362-028X.JFP-11-479 23043825

[B9] Schwaiger K Huther S Holzel C Kampf P Bauer J Prevalence of antibiotic-resistant enterobacteriaceae isolated from chicken and pork meat purchased at the slaughterhouse and at retail in Bavaria, Germany Int J Food Microbiol 2012 154 3 206 211 10.1016/j.ijfoodmicro.2011.12.014 22260925

[B10] Koldovsky O Kong W Philipps AF Rao RK Studies on milk-borne insulin-like growth factor-1 and 2 (IGF-1 and IGF-2) and epidermal growth factor (EGF) in suckling rats Endocr Regul 1993 27 3 149 153 8193315

[B11] Blum JW Baumrucker CR Insulin-like growth factors (IGFs), IGF binding proteins, and other endocrine factors in milk: role in the newborn Adv Exp Med Biol 2008 606 397 422 10.1007/978-0-387-74087-4_16 18183939

[B12] Jaeger LA Lamar CH Cline TR Cardona CJ Effect of orally administered epidermal growth factor on the jejunal mucosa of weaned pigs Am J Vet Res 1990 51 3 471 474 2107778

[B13] Fagbemi AO Wright N Lakhoo K Edwards AD Immunoreactive epidermal growth factor receptors are present in gastrointestinal epithelial cells of preterm infants with necrotising enterocolitis Early Hum Dev 2001 65 1 1 9 10.1016/S0378-3782(01)00164-5 11520624

[B14] Clark JA Lane RH Maclennan NK Holubec H Dvorakova K Halpern MD Williams CS Payne CM Dvorak B Epidermal growth factor reduces intestinal apoptosis in an experimental model of necrotizing enterocolitis Am J Physiol Gastrointest Liver Physiol 2005 288 4 G755 G762 10.1152/ajpgi.00172.2004 15528252

[B15] Nakai K Hamada Y Kato Y Kitagawa K Hioki K Ito S Okumura T Further evidence that epidermal growth factor enhances the intestinal adaptation following small bowel transplantation Life Sci 2004 75 17 2091 2102 10.1016/j.lfs.2004.04.018 15312753

[B16] Rao RK Koldovsky O Grimes J Williams C Davis TP Regional differences in gastrointestinal processing and absorption of epidermal growth factor in suckling rats Am J Physiol 1991 261 5 Pt 1 G790 G798 1951698 10.1152/ajpgi.1991.261.5.G790

[B17] Rao RK Luminal processing of epidermal growth factor in mouse gastrointestinal tract in vivo Peptides 1995 16 3 505 513 10.1016/0196-9781(95)00002-2 7651906

[B18] Shen WH Xu RJ Stability of epidermal growth factor in the gastrointestinal lumen of sucking and weaned pigs Life Sci 1996 59 3 197 208 10.1016/0024-3205(96)00285-8 8699930

[B19] Rao RK Baker RD Baker SS Bovine milk inhibits proteolytic degradation of epidermal growth factor in human gastric and duodenal lumen Peptides 1998 19 3 495 504 10.1016/S0196-9781(97)00462-2 9533637

[B20] Shen WH Xu RJ Stability and distribution of orally administered epidermal growth factor in neonatal pigs Life Sci 1998 63 10 809 820 10.1016/S0024-3205(98)00337-3 9734700

[B21] Playford RJ Hanby AM Gschmeissner S Peiffer LP Wright NA McGarrity T The epidermal growth factor receptor (EGF-R) is present on the basolateral, but not the apical, surface of enterocytes in the human gastrointestinal tract Gut 1996 39 2 262 266 10.1136/gut.39.2.262 8977341 PMC1383309

[B22] Thompson JF Specific receptors for epidermal growth factor in rat intestinal microvillus membranes Am J Physiol 1988 254 3 Pt 1 G429 G435 3348408 10.1152/ajpgi.1988.254.3.G429

[B23] Herbst RS Review of epidermal growth factor receptor biology Int J Radiat Oncol Biol Phys 2004 59 2 Suppl 21 26 10.1016/j.ijrobp.2003.11.041 15142631

[B24] Oda K Matsuoka Y Funahashi A Kitano H A comprehensive pathway map of epidermal growth factor receptor signaling Mol Syst Biol 2005 1 2005 0010 10.1038/msb4100014 16729045 PMC1681468

[B25] James PS Smith MW Tivey DR Wilson TJ Epidermal growth factor selectively increases maltase and sucrase activities in neonatal piglet intestine J Physiol 1987 393 583 594 3328784 10.1113/jphysiol.1987.sp016842 PMC1192412

[B26] Lee DN Kuo TY Chen MC Tang TY Liu FH Weng CF Expression of porcine epidermal growth factor in Pichia pastoris and its biology activity in early-weaned piglets Life Sci 2006 78 6 649 654 10.1016/j.lfs.2005.05.067 16111721

[B27] Kang P Toms D Yin Y Cheung Q Gong J De Lange K Li J Epidermal growth factor-expressing Lactococcus lactis enhances intestinal development of early-weaned pigs J Nutr 2010 140 4 806 811 10.3945/jn.109.114173 20147464

[B28] Buret A Olson ME Gall DG Hardin JA Effects of orally administered epidermal growth factor on enteropathogenic Escherichia coli infection in rabbits Infect Immun 1998 66 10 4917 4923 9746597 10.1128/iai.66.10.4917-4923.1998 PMC108608

[B29] Cheung QC Yuan Z Dyce PW Wu D DeLange K Li J Generation of epidermal growth factor-expressing Lactococcus lactis and its enhancement on intestinal development and growth of early-weaned mice Am J Clin Nutr 2009 89 3 871 879 10.3945/ajcn.2008.27073 19176742

[B30] Madsen SM Arnau J Vrang A Givskov M Israelsen H Molecular characterization of the pH-inducible and growth phase-dependent promoter P170 of Lactococcus lactis Mol Microbiol 1999 32 1 75 87 10.1046/j.1365-2958.1999.01326.x 10216861

[B31] Mierau I Kleerebezem M 10 years of the nisin-controlled gene expression system (NICE) in Lactococcus lactis Appl Microbiol Biotechnol 2005 68 705 717 10.1007/s00253-005-0107-6 16088349

[B32] Kuipers OP Beerthuyzen MM de Ruyter PG Luesink EJ de Vos WM Autoregulation of nisin biosynthesis in Lactococcus lactis by signal transduction J Biol Chem 1995 270 45 27299 27304 10.1074/jbc.270.45.27299 7592991

[B33] Bermudez-Humaran LG Langella P Commissaire J Gilbert S Le Loir Y L’Haridon R Corthier G Controlled intra- or extracellular production of staphylococcal nuclease and ovine omega interferon in Lactococcus lactis FEMS Microbiol Lett 2003 224 2 307 313 10.1016/S0378-1097(03)00475-0 12892897

[B34] Xu S Linher-Melville K Yang BB Wu D Li J Micro-RNA378 (miR-378) regulates ovarian estradiol production by targeting aromatase Endocrinology 2011 152 10 3941 3951 10.1210/en.2011-1147 21846797 PMC3176644

[B35] Petersen YM Elnif J Schmidt M Sangild PT Glucagon-like peptide 2 enhances maltase-glucoamylase and sucrase-isomaltase gene expression and activity in parenterally fed premature neonatal piglets Pediatr Res 2002 52 4 498 503 10.1203/00006450-200210000-00007 12357042

[B36] Lowry OH Rosebrough NJ Farr AL Randall RJ Protein measurement with the Folin phenol reagent J Biol Chem 1951 193 1 265 275 14907713

[B37] Torrallardona D Conde MR Badiola I Polo J Brufau J Effect of fishmeal replacement with spray-dried animal plasma and colistin on intestinal structure, intestinal microbiology, and performance of weanling pigs challenged with Escherichia coli K99 J Anim Sci 2003 81 5 1220 1226 12772849 10.2527/2003.8151220x

[B38] Cera KR Mahan DC Cross RF Reinhart GA Whitmoyer RE Effect of age, weaning and postweaning diet on small intestinal growth and jejunal morphology in young swine J Anim Sci 1988 66 2 574 584 3372395 10.2527/jas1988.662574x

[B39] Nabuurs MJ Weaning piglets as a model for studying pathophysiology of diarrhea Vet Q 1998 20 Suppl 3 S42 S45 10.1080/01652176.1998.9694967 9689724

[B40] Kiarie E Nyachoti CM Slominski BA Blank G Growth performance, gastrointestinal microbial activity, and nutrient digestibility in early-weaned pigs fed diets containing flaxseed and carbohydrase enzyme J Anim Sci 2007 85 11 2982 2993 10.2527/jas.2006-481 17686904

[B41] Dignass AU Sturm A Peptide growth factors in the intestine Eur J Gastroenterol Hepatol 2001 13 7 763 770 10.1097/00042737-200107000-00002 11474304

[B42] Choi HJ Ahn JH Park SH Do KH Kim J Moon Y Enhanced wound healing by recombinant Escherichia coli Nissle 1917 via human epidermal growth factor receptor in human intestinal epithelial cells: therapeutic implication using recombinant probiotics Infect Immun 2012 80 1079 1087 10.1128/IAI.05820-11 22184415 PMC3294649

[B43] Chandra A Lan S Zhu J Siclari VA Qin L Epidermal growth factor receptor (EGFR) signaling promotes proliferation and survival in osteoprogenitors by increasing early growth response 2 (EGR2) expression J Biol Chem 2013 288 20488 20498 10.1074/jbc.M112.447250 23720781 PMC3711314

[B44] Wallace LE Hardin JA Gall DG Expression of EGF and erbB receptor proteins in small intestinal epithelium Can J Gastroenterol 2001 15 suppl SA abstr 135

[B45] Kelly D McFadyen M King TP Morgan PJ Characterization and autoradiographic localization of the epidermal growth factor receptor in the jejunum of neonatal and weaned pigs Reprod Fertil Dev 1992 4 2 183 191 10.1071/RD9920183 1438948

[B46] Schweiger M Steffl M Amselgruber WM Differential expression of EGF receptor in the pig duodenum during the transition phase from maternal milk to solid food J Gastroenterol 2003 38 7 636 642 10.1007/s00535-002-1115-2 12898355

[B47] Ginneken CV Haver EV Oste M Weyns A The presence of EGF- and IGF-1-receptors in the smallintestine of fetal, neonatal and weaned piglets Livest Sci 2007 108 57 60 10.1016/j.livsci.2007.01.054

[B48] Miettinen PJ Perheentupa J Otonkoski T Lahteenmaki A Panula P EGF- and TGF-alpha-like peptides in human fetal gut Pediatr Res 1989 26 1 25 30 10.1203/00006450-198907000-00009 2788859

[B49] Ulshen MH Lyn-Cook LE Raasch RH Effects of intraluminal epidermal growth factor on mucosal proliferation in the small intestine of adult rats Gastroenterology 1986 91 5 1134 1140 3489652 10.1016/s0016-5085(86)80008-7

[B50] Abud HE Watson N Heath JK Growth of intestinal epithelium in organ culture is dependent on EGF signalling Exp Cell Res 2005 303 2 252 262 10.1016/j.yexcr.2004.10.006 15652340

[B51] Roy M King TW Epidermal growth factor regulates NIKS keratinocyte proliferation through Notch signaling J Surg Res 2013 185 6 11 10.1016/j.jss.2013.06.046 23899513

[B52] Bhandari SK Xu B Nyachoti CM Giesting DW Krause DO Evaluation of alternatives to antibiotics using an Escherichia coli K88+ model of piglet diarrhea: effects on gut microbial ecology J Anim Sci 2008 86 4 836 847 10.2527/jas.2006-822 18192551

[B53] Thu TV Loh TC Foo HL Yaakub H Bejo MH Effects of liquid metabolite combinations produced by Lactobacillus plantarum on growth performance, faeces characteristics, intestinal morphology and diarrhoea incidence in postweaning piglets Trop Anim Health Prod 2011 43 1 69 75 10.1007/s11250-010-9655-6 20632092 PMC2995859

[B54] May KD Wells JE Maxwell CV Oliver WT Granulated lysozyme as an alternative to antibiotics improves growth performance and small intestinal morphology of 10-day-old pigs J Anim Sci 2012 90 4 1118 1125 10.2527/jas.2011-4297 22064735

[B55] Caspary WF Physiology and pathophysiology of intestinal absorption Am J Clin Nutr 1992 55 1 Suppl 299S 308S 1728844 10.1093/ajcn/55.1.299s

[B56] Lee DN Chang WF Yu IT Chiou WS Weng CF Effects of diets supplemented with recombinant epidermal growth factor and glutamine on gastrointestinal tract development of early-weaned piglets Asian-Aust J Anim Sci 2008 21 582 589 10.5713/ajas.2008.70181

[B57] Resta-Lenert S Barrett KE Live probiotics protect intestinal epithelial cells from the effects of infection with enteroinvasive Escherichia coli (EIEC) Gut 2003 52 7 988 997 10.1136/gut.52.7.988 12801956 PMC1773702

[B58] Marteau P Rambaud JC, Buts JP, Cortier G, Flourié B Factor Controlling the Bacterial Microflora Gut Microflora: Digestive Physiology and Pathology 2006 1 J Libbey Eurotext, DL, Paris, France 37 55

[B59] Servin AL Coconnier MH Adhesion of probiotic strains to the intestinal mucosa and interaction with pathogens Best Pract Res Clin Gastroenterol 2003 17 5 741 754 10.1016/S1521-6918(03)00052-0 14507585

